# Assessing knowledge, attitude, and practice of emergency contraception: a cross- sectional study among Ethiopian undergraduate female students

**DOI:** 10.1186/1471-2458-12-110

**Published:** 2012-02-09

**Authors:** Fatuma A Ahmed, Kontie M Moussa, Karen O Petterson, Benedict O Asamoah

**Affiliations:** 1 International Master Programme in Public Health, Faculty of Medicine, Lund University, Malmö University Hospital, Malmö, Sweden; 2 Department of Clinical Sciences Malmö, Division of Social Medicine and Global Health, Lund University, Malmö University Hospital, Malmö, Sweden

**Keywords:** Emergency contraception, Knowledge, Attitude, Practice, Addis Ababa University, Ethiopia

## Abstract

**Background:**

Emergency contraception (EC) is a type of modern contraception which is indicated after unprotected sexual intercourse when regular contraception is not in use. The importance of EC is evident in preventing unintended pregnancies and its ill consequences like unintended child delivery or unsafe abortion, which are the most common causes of maternal mortality. Therefore, EC need to be available and used appropriately as a backup in case regular contraception is not used, misused or failed. Knowing that Ethiopia is one of the countries with highest maternal mortality rate, this study aimed to assess the knowledge, attitude and practice of EC, and to further elucidate the relationship between these factors and some socioeconomic and demographic characteristics among female undergraduate students of Addis Ababa University (AAU). This information will contribute substantially to interventions intended to combat maternal mortality.

**Methods:**

A Cross-sectional quantitative study among 368 AAU undergraduate students was conducted using self-administered questionnaire. Study participants were selected by stratified random sampling. Data was entered and analyzed using SPSS Version 17. Results were presented using descriptive statistics, cross-tabulation and logistic regression.

**Results:**

Among the total participants (n = 368), only 23.4% were sexually active. Majority (84.2%) had heard of EC; 32.3% had a positive attitude towards it. The main source of information reported by the respondents was Media (69.3%). Among those who were sexually active, about 42% had unprotected sexual intercourse. Among those who had unprotected sexual intercourse, 75% had ever used EC. Sexually active participants had significantly better attitude towards EC than sexually inactive participants (crude OR 0.33(0.15-0.71)); even after adjusting for possible confounders such as age, region, religion, ethnicity, marital status, department and family education and income (adj. OR 0.36(0.15-0.86)).

**Conclusions:**

The study showed high EC awareness and usage in contrast to other studies in the city, which could be due to the fact that university students are relatively in a better educational level. Therefore, it is highly recommended that interventions intended to combat maternal mortality through contraceptive usage need to be aware of such information specific to the target groups.

## Background

EC is a type of modern contraception which is indicated after unprotected sexual intercourse, following sexual abuse, misuse of regular contraception or non use of contraception [1]. EC plays a vital role in preventing unintended pregnancy, which in turn helps to reduce unintended child birth and unsafe abortion, which are major problems of maternal health [1]. EC is found to be effective if used as soon as possible after unprotected sexual intercourse, especially within 72 hours of unprotected sexual intercourse [2].

There are two types of ECs namely, emergency contraceptive pills and intrauterine devices (IUDs). The pills include combined oral contraceptive pills (COCs), and a progestin only pills (POPs); IUDs can be effective if it is inserted within 5 days of unprotected sexual intercourse [3]. EC is said to be safe with minor side effects like nausea and vomiting in case of pills and infection for IUDs if not used properly [3]. Effectiveness of EC said to be 75% in case of COCs and 85% in case of POPs [4]. Regarding the mechanism of action, EC works by preventing fertilization, implantation and tubal transportation of sperm and ovum [4].

Each year there are about 250 Million pregnancies globally and one third of these are unintended and 20% of these undergo induced abortion [5]. In Low income countries, more that one third of the 182 million pregnancies is unintended; the fate of 19% will be induced abortion and 11% of this is unsafe [5]. In low income countries, the women who do not use any contraceptive contribute to two third of unintended pregnancies, where more than 100 million married women have unmet need for contraception [5]. Unsafe abortion has much ill effects in women's health, each year about 68,000 women die because of unsafe abortion, and millions of women end up with many complications of unsafe abortion, which could include severe infection and bleeding; this could have been immensely reduced by using EC [6]. Each year about 500,000 women die due to cases related with child birth, and majority are in sub Saharan Africa where there is also high fertility rate that is more than five [7]. Globally, it's estimated that 11% births are given by adolescent girls of age 15-19 annually, and 95% of these births are in low income countries, Ethiopia is one of the countries with high adolescent birth rate [8]. Most adolescent pregnancies seem to be intended; just because they happen within marriage but in reality most of them are unintended rather the marriage itself is arranged by the girls' family due to some cultural influences [8]. Adolescent pregnancy affect the health of mother and child, it has a devastating impact in social and psychological life of the girls [8].

Ethiopia is one of the countries with high maternal mortality rate; the estimated rate in 2005 was 673 per 100,000 live births [9]. In one of the surveys conducted in Ethiopia, among 1075 women who presented with abortion, about 58% were between age group of 20-29; and non use of contraceptive contributed to 78% of these pregnancies and rape accounted for 3% of the abortions [10]. In a study conducted in one of the high schools of Ethiopia, the prevalence of attempted rape was 8.8% and the prevalence of performed rape was 11.5% [11].

Despite the fact that different modern contraceptives exist world wide, the problem of unintended pregnancy still exists, which could be due to gap in awareness, negative attitudes towards contraception, low accessibility or as a result of sexual assault. At times, the knowledge and practice might be there but no contraceptive is 100% effective, and it is always very vital to have EC as a backup method. In one of the studies conducted among 417 women of post abortal care clients in Ethiopia, 59(14.1%) had ever heard of EC, and only 15(8.6%) had ever used EC [12]. In another study among 833 college students in one of the towns of Ethiopia, the magnitude of sexual violence was 47.9%, and unwanted pregnancy was found to be 16.9%; about 228(27.4%) had knowledge about EC, 20(2.4%) had ever used it and about 548(65.8%) had favorable attitude towards use of EC [13].

Considering the importance of EC in preventing unintended pregnancy, this study aimed to assess the knowledge, attitude and practice of EC and to further elucidate the relationship between these factors and socioeconomic and demographic characteristics among female undergraduate students of AAU.

## Methods

### Study area

The study was conducted in AAU, the biggest University of Ethiopia, which is located in the capital city of the country, Addis Ababa. Ethiopia is one of the low income countries in sub Saharan Africa, with high fertility rate, which is about 5.4 [14]. In Ethiopia, there are different ethnic groups with different cultures, for example, Amhara, Oromo, and Tigre etc. Christianity and Islam are the major religions in the country [14].

Student in this University come from different regions of the country, and this created a favorable condition to compare the results among students of different regions. According to the registrar's office of the University, the total number of students enrolled at the time of the study was about 21, 819 undergraduate students, among these about 6725 were female [unpublished data].

### Study period

The study was conducted from the beginning of February, 2010 till the beginning of May, 2010.

### Study design

A Cross- sectional quantitative study was conducted.

### Source population

The source population for this study was all female undergraduate students at AAU. According to AAU registrar's office, there were about 6725 female undergraduate students in 2010; 884(13%) of them were admitted to health science departments and 5841(87%) were admitted to non- health science departments.

### Sampling technique

To obtain a representative sample, stratified random sampling was applied to select study participants from the source population. First the students were divided in to two practical strata, which were health science students and non-health science students. From each stratum, participants were selected by simple random sampling based on the proportion of the number of female students in each stratum that is 13% health science students and 87% non- health science students. All female undergraduate students at the University were eligible for the study.

### Sample size

The minimum required sample size was calculated using electronic sample size calculator (with 5% margin of error, 95% confidence level and 50% response distribution); and it was found to be 400(including 10% non-response rate). The total number of students who answered the questionnaire was 368, making the response rate of the study 92%.

### Data collection method

Data was collected using self- administered Questionnaire which [additional file 1] was prepared in English to assess socioeconomic status, reproductive characteristics as well as their knowledge, attitude and practice towards EC. To increase the quality of the data, most of the questions were adapted from previously conducted studies with some changes based on the local context [15]. Likewise, confidentiality and anonymity of the study was reassured. The data was collected while students were in class rooms. The instructors cooperated with the principal investigator in disseminating the questionnaire. In the end, the questionnaires were gathered and checked for completeness by the principal investigator.

### Ethical consideration

Before the data was collected, official letter from Lund University administration was obtained to ask consent from AAU administration as well as from study participants. The purpose of the study was explained to all study participants; they were also informed that all of their responses are confidential and anonymous, and they have all the right not to be involved in the study or not to answer any of the questions. Ethical approval was issued by Addis Ababa city administration health bureau.

### Data analysis

Data was entered and analyzed using SPSS version 17 by the principal investigator. Different forms of analysis like descriptive statistics, cross tabulation and logistic regression were applied to present the results. Recoding of data was also done for some variables to fit them in to binary logistic regression model. Adequate time was spent on the analysis to ensure quality.

### Study variables

#### Dependent variables

Knowledge, attitude and practice of EC. These variables have been defined below under "operational definitions".

#### Independent variables

• **Age (in years): **different age groups: 17-20, 21-23, 24-26, 27-29.

• **Religion: **Orthodox Christian, Protestant, Muslim, and Others (Catholic, 7th Adventist).

• **Ethnicity: **Amhara, Oromo, Tigre, and others (Somalia, SNNP, Affar)

• **Region: **Addis Ababa, Oromo, Amhara, SNNP, Tigri, Harar, Affar

• **Department: **Health science and non-health science

• **Marital status: **Unmarried, married, divorced and widowed.

• **Family monthly income (in birr): **less than 150, 150-249,250-499,500-999, 1000-1499, more than 1500.

• **Parents' educational status: **Can't write and read, can write and read, primary school (1-8th), secondary school (9-12th), and Higher education.

### Operational definitions

• **Emergency contraception: **A kind of contraception indicated after unprotected sexual intercourse to prevent unintended pregnancy.

• **Sexually active: **having a previous history of vaginal sexual intercourse.

• **Unintended pregnancy: **pregnancy occurred with no plan.

• **Knowledge: **awareness of the existence of EC, its importance and effectiveness.

• **Attitude: **Intention of using or recommending EC when a need arises. Intending to use or recommend is considered as a positive attitude, and no intention as a negative attitude.

• **Practice: **Any previous history of EC usage.

• **High income: **Monthly income of more than 500 Ethiopian birr (33 USD)

• **Low income: **Monthly income of 150 to 499 Ethiopian Birr (10-32 USD)

• **Media: **Radio, Television.

## Results

### Socio demographic characteristics

In this study, a total of 368 students answered the questionnaire (92% response rate). As shown in Table 1, the age range of study participants were from 17 to 29 years old, making the mean and standard deviation of 20.5 and 1.75 respectively. Majority (57.1%) was between the age group 17 to 20 years; 37% between 20 to 23; 4.1% between 23-26; and those between 26-29 years were 7(1.9%). Majority of the respondents 232(63%) were Orthodox Christians by religion, followed by Protestant Christians 79 (21.5%), and Muslims 49(13.3%). Regarding their ethnicity, Amhara was 176(47.%); Oromo, 85(23.1%); Tigre, 44(12%); the rest (17.1%) accounted for SNNP, Somalia, and Afar. Majority of the students (45.1%) had been living in Addis Ababa before entering to the university; the rest had been living in other different regions of Ethiopia. Most of the students (90.5%) were unmarried. Their family income ranged from less than 150 birr which accounts for 6(1.6%) of the students, to more than 1500 birr which were about 163(44.3%) of the students. About 51(13.9%) of the students responded that their mothers can't write and read; and about 102(27.7%) reported that their mothers attended higher education. About their father's education, 155(42.1%) reported their fathers attended higher education, and about 19(5.2%) responded that their fathers can't write and read.

**Table 1:** Socio demographic characteristics of undergraduate female students of AAU, Ethiopia, March, 2010(n = 368)

Variable	Number	Percentage
**Age**		
17-20	210	57.0
20-23	136	37.0
23-26	15	4.1
26-29	7	1.9

**Religion**		
Orthodox	232	63.0
Muslim	49	13.3
Protestant	79	21.5
Other(Adventist, Catholic)	8	2.2

**Ethnicity**		
Amhara	176	47.8
Oromo	85	23.1
Tigre	44	12.0
Other(Somali, Afar, SNNP)	63	17.1

**Marital Status**		
Non- married	339	92.1
Married	29	7.9

**Family monthly income**		
< 150	6	1.6
150-1499	199	54.1
> 1500	163	44.3

**Mother's educational status^1^**		
Can't write and read	51	13.9
Can write and read	85	23.1
Primary School and above(1-8^th ^grade)	231	62.8

**Father's educational Status**^2^		
Can't write and read	19	5.2
Can write and read	63	17.1
Primary School and above	282	76.6

^1^Missing Values: 1(0.33%)

^2^Missing Values: 4(1.1%)

### Reproductive characteristics

As presented in Table 2, the majority of the study subjects 282(76.6%) were sexually inactive. About 36(9.8%) had history of unprotected sexual intercourse, this was 42% when calculated only among sexually active participants; and 13(3.5%) experienced unintended pregnancy. Regarding the reasons for the unintended pregnancy, 3(0.8%) responded it was due to pressure from the partner, 2(0.5%) by forced sexual intercourse, 2(0.5%) reported it was because they forgot to take contraceptive, 1.6% was due to contraceptive failure.

**Table 2:** Reproductive characteristics of female undergraduate students of AAU, Ethiopia, March 2010 (n = 368)

Variable	Number	Percent
**Sexual Activity**		
Yes	86	23.4
No	282	76.6

**Unprotected sexual intercourse**^1^		
Yes	36	9.8
No	50	13.5

**Number of Children**^2^		
Zero	69	18.74
One	13	3.5
Two	4	1.1

**Unintended Pregnancy**^3^		
Yes	13	3.5
No	73	19.83

**Reason for unintended Pregnancy**^4^		
Contraceptive failure	6	1.6
Forget to take contraceptive	2	0.5
Pressure from partner	3	0.8
Forced to have sex	2	0.5

^1^Only those who are sexually active answered the questions

^2^Only those who are sexually active answered the questions

^3^Only those who are sexually active answered the questions

^4^Only those who had unintended pregnancy answered the question

### Knowledge, attitude and practice of EC

As it is noted in Table 3, 310(84.2%) had ever heard of EC; and a greater number (336) had ever heard of other contraception. Among the respondents who had ever heard of EC, 85(23.1%) reported that it is 99% effective and 216(58.7%) responded that it is safe to use. Regarding their source of information about EC, 278(75.5%) reported Media, 255(69.3%) health facilities, 108(29.3%) formal education, 29(7.9%) Internet, 15(4.1%) magazine, and 7(1.9) heard from a friend or a relative. Majority of the students (78.3%) responded that ECs are obtained from health institutes, and 302(82.1%) mentioned that EC helps during post rape, 182(49.5%) knew it is worth as a back up when condom breaks, 41(11.1%) thought it is important when oral contraceptive pill (OCP) is forgotten. About the health risks of EC, 49(13.3%) thought it can cause health problems, 33(9%) said it can hurt if it doesn't work, 28(7.6%) reported that it can result in sexually transmitted infection (STI) and Human immuno deficiency virus (HIV) infection. Those respondents who had an intention to use or recommend EC for a friend when a need arises were 119(32.3%). Among those who had unprotected sexual intercourse,75% had ever used EC, that was 30.7% of those who were sexually active and ever heard of EC, and that was only 7.3% of the total participants. Among those who had ever used EC, 15 of them reported that it was recommended by health professional and the rest by a friend. Among the total participants, 48(13%) had ever used other contraception, that was 37.8% of sexually active students that ever heard of other contraception.

**Table 3:** Knowledge, attitude and practice of emergency contraception among undergraduate AAU students, March 2010 (n = 368)

Variable	Number	Percentage
**Ever heard of EC**		
Yes	310	84.2
No	58	15.8

**Time heard about EC**		
< 6 Months ago	31	8.4
6 months-5 years	183	49.7
Before 5 years	131	35.6

**Time to take EC**^1^		
Within 72 hours	237	64.4
After 72 hours	21	5.7
Don't know	67	18.2

**Effectiveness of EC**^2^		
75-99%	186	50.5
30-50%	34	9.3
Not sure	102	l27.7

**How safe is EC**^3^		
Safe	216	58.7
Unsafe	19	5.2
I don't know	87	23.6

**Source of information**^♣^		
Formal Education	108	29.3
Media	278	75.5
Magazine	15	4.1
Internet	29	7.9
Health facilities	255	69.3
Friends/relatives	7	1.9

**Place to obtain EC**		
Health institutes	288	78.3
Supermarket	2	0.5
Social worker	2	0.5
Don't know	2	0.5
Impossible to obtain	13	3.5

**Importance of EC**^♠^		
Post rape	302	82.1
Back up when condom breaks	182	49.5
When oral contraceptive pill is forgotten	41	11.1

N.B: Only those who had ever heard of emergency contraceptive answered other knowledge questions.

Table 4 presents that there is no statistically significant association between students' awareness about the existence of EC and their socio demographic characteristics.

**Table 4:** Association between EC awareness and sociodemographic status of female undergraduate students of AAU, March, 2010(n = 368)

Variables	Awareness		95% Crude OR	95% CI Adjusted OR
			
	Yes	No		
**Age**				
26-29	6	1	1.0	1.0
23-26	13	2	0.92(0.06-12.28)	0.66(0.04-9.73)
20-23	115	21	1.09(0.12-9.57)	0.90(0.09-8.93)
17-20	176	34	1.15(0.13-9.93)	0.88(0.09-8.65)

**Religion**				
Muslim	37	12	1.0	1.0
Christian	273	46	1.92(0.93-3.96)	0.26(0.04-1.53)

**Department**				
Health science	45	3	1.0	1.0
Non Health Science	265	55	0.32(0.09-1.07)	7.85(0.84-73.39)

**Region**				
Addis Ababa	143	23	1.0	1.0
Out of Addis Ababa	167	35	0.76(0.43-1.35)	1.35(0.43-4.24)

**Marital Status**				
Married	25	4	1.0	1.0
Non-Married	285	54	0.84(0.28-2.52)	0.56(0.14-2.14)

**Family Monthly Income**				
> 500	253	47	1.0	1.0
150-499	57	11	1.03(0.50-2.12)	1.18(0.23-5.92)

**Mother's education**				
> 8th grade	145	26	1.0	1.0
< 8th grade	164	32	1.08(0.61-1.91)	2.046(0.47-8.87)

**Father's education**				
> 8th grade	197	37	1.0	1.0
< 8th grade	110	20	0.96(0.53-1.75)	0.77(0.34-1.74)

**N.B**. Adjusted OR of each variable is obtained after adjusting it for all other variables shown in the table.

In Table 5, it is shown that those participants with low family income had a better attitude than those with high family income (crude OR 2.12(1.06-4.18)); this significant association remained when it was adjusted for age, region, religion, ethnicity, marital status, and family education. (Adj.0R 2.47(1.06-5.78)).

**Table 5:** Association between EC attitude and sociodemographic status of female undergraduate students of AAU, March, 2010(n = 368)

Variables	Attitude		95% CI Crude OR	95% CI adjusted OR
			
	positive	negative		
**Age**				
26-29	5	1	1.0	1.0
23-26	8	2	1.25(0.08-17.65)	1.37(0.08-21.22)
20-23	44	22	2.50(0.27-22.72)	2.80(0.27-28.45)
17-20	52	55	4.43(0.50-39.14)	4.22(0.42-41.73)

**Religion**				
Muslim	11	11	1.0	1.0
Christian	108	69	1.56(0.64-3.80)	1.89(0.70-5.15)

**Department**				
Health science	26	13	1.0	1.0
Non Health Science	93	67	0.69(0.33-1.44)	0.95(0.43-2.09)

**Region**				
Addis Ababa	53	34	1.0	1.0
Out of Addis Ababa	66	46	0.92(0.51-1.63)	1.07(0.56-2.04)

**Marital Status**				
Married	13	4	1.0	1.0
Non-Married	106	76	0.42(0.13-1.36)	0.72(0.18-2.74)

**Family Monthly Income**				
> 500	99	56	1.0	1.0
150-499	20	24	2.12(1.07-4.17)*	2.47(1.06-5.78)**

**Mother's education**				
> 8th grade	57	33	1.0	1.0
< 8th grade	61	47	1.33(0.75-2.36)	1.34(0.58-3.08)

**Father's education**				
> 8th grade	76	52	1.0	1.0
< 8th grade	41	27	0.96(0.52-1.75)	0.57(0.24-1.36)

*statistically significant

** Statistically significant after adjusted for other variables shown on the table.

In Table 6, it is noted that there is no statistically significant association between respondents' socio- demographic characteristics and usage of EC.

**Table 6:** Association between EC usage and socidemographic status of female undergraduate students of AAU, March, 2010(n = 368)

Variables	Usage		95% CI Crude OR	95% CI Adjusted OR
			
	Yes	No		
**Age**				
26-29	2	3	1.0	1.0
23-26	2	9	3.00(0.28-31.63)	3.57(0.22-56.58)
20-23	11	27	1.63(0.24-11.18)	1.89(0.15-22.60)
17-20	12	21	1.16(0.17-7.99)	0.92(0.07-11.71)

**Religion**				
Christian	23	57	1.0	1.0
Muslim	4	2	0.41(0.09-1.78)	0.26(0.04-1.53)

**Department**				
Health science	1	11	1.0	1.0
Non Health Science	26	50	5.83(0.71-47.74)	7.85(0.84-73.39)

**Region**				
Addis Ababa	7	20	1.0	1.0
Out of Addis Ababa	20	41	1.32(0.47-3.66)	1.35(0.43-4.24)

**Marital Status**				
Married	7	15	1.0	1.0
Non-Married	20	46	0.87(0.30-2.48)	0.56(0.14-2.14)

**Family Monthly Income**				
> 500	23	52	1	1
150-499	4	9	1.01(0.28-3.63)	1.18(0.23-5.92)

**Mother's education**				
> 8th grade	13	29	1.0	1.0
< 8th grade	14	31	0.96(0.38-2.389)	2.04(0.472-8.872)

**Father's education**				
> 8th grade	17	43	1.0	1.0
< 8th grade	10	18	0.67(0.25-1.75)	0.38(0.09-1.62)

**N.B: **Only sexually active participants who ever heard of EC are included.

**: **Adjusted OR of each variable is obtained after adjusting it for all other variables shown in the table.

Table 7 shows that those who were sexual active had a better attitude than sexually inactive participants (crude OR 0.33(0.15-0.71)); this remained significantly associated when it was adjusted for age, region, religion, ethnicity, marital status, department and family education and income (adj. OR 0.36(0.15-0.86)).

**Table 7:** Association of EC knowledge, attitude, and practice with sexual activity, AAU female undergraduate students, March, 2010

Variables	Sexual activity		95% CI Crude OR	95% CI Adjusted OR
	**Yes**	**No**		

**Heard of EC**				
Yes	77	233	1.0	1.0
No	9	49	0.55(0.26-1.18)	0.42(0.17-1.03)

**Attitude**				
Positive	36	83	1.0	1.0
Negative	10	70	0.33(0.15-0.71)*	0.36(0.15-0.86)**

**Usage**				
Yes	27	0	1.0	1.0
No	55	6	ns	ns^β^

*statistically significant

** remained statistically significant when adjusted with age, region, religion, department, marital status, family income and education

^β ^ns: non-significant(OR O.00(0.00-

## Discussion

As we noted from the result of the actual study, participants who had ever heard of EC were 84.2%; those who had positive attitude towards it were 32.3%. Only 23.4% of the participants were sexually active, and 42% these had unprotected sexual intercourse. Among those who had unprotected sexual intercourse, 75% had ever used EC, which was 30.7% of those who were sexually active and ever heard of EC, and that was only 7.3% of the total participants. There seem to be low usage of EC in the study population due to the high proportion of sexually inactive participants. When the sample was stratified, it was realized that usage of EC was actually high among the sexually active participants.

Awareness of EC is relatively higher among the participants of this study than other similar studies conducted in Addis Ababa. For example, in a study conducted in 2006 among 636 antenatal care clients of selected Addis Ababa health centers, the women who were aware of EC were only 10.2% and those who had positive attitude were 37.6% [15]. A similar study was conducted in the Western Cape province of South Africa, among 831 sexually active women in selected public clinic; those who were aware of EC were only 30% [16]. In 2009, there was also a similar study conducted among 300 women of age 18-45 years old at Lyari general hospital at Karachi, about 48% of the respondents had ever heard of EC [17].

This variation seems to be due to the difference in their level of education, which can have an influence on the awareness level of EC. It is believed that educated people are much more dedicated to their health than non-educated people; and in most of the cases, they can have a tendency to gather information in this regard.

In this study, Media was found to be the main source of information for EC. In a similar study conducted in Nigeria among undergraduate college students, the main source of information was through friends/peers [18]. In another study conducted in 2005 among 379 female students of Makerere University of Uganda, only 45.1% had ever heard of EC; their main source of information were also via friends 34% followed by Media 24.8% [19].

In the above mentioned study conducted in 2006 among antenatal care clients in Addis Ababa, there were some association between economic status and awareness of EC, those with higher income had a better attitude. Unexpectedly, in this study those with lower family income had a better attitude; this could be explained due to the fact that the main source of information for EC in this study was media which can be accessed by most students in the university regardless of their economic status. In this study, the absence of significant association between attitude and their ethnicity as well as between attitude and religion can be explained by the fact that these study subjects share the same cultural values due to their social interaction in the campus, which influences them to have more or less the same attitude towards EC. In this study, sexually active participants had a better attitude than sexually inactive participants, who are believed to be reluctant about EC.

In this study one important aspect we need to give attention to, is the presence of sexual abuse; about 5 students reported that the reason for their unintended pregnancy was due to forced sexual intercourse.

It is assumed that the findings of this study can be generalized because of the representative nature of study participants (random sampling) and adequate number of sample size. Although the questionnaire contained some socially sensitive questions, in order to obtain a reliable data, respondents were well informed about the purpose of the study and they were reassured about confidentiality and anonymity. Conducting the study among these study participants who are relatively in higher educational level is novel about the study since this is a good opportunity to elucidate the influence of education in awareness of EC.

## Conclusions

The study showed high level of EC awareness and usage in contrast to other studies in the city; this could be due to the fact that these study participants are relatively in higher educational level in comparison to other women in the city. However, it was shown that there was low level of positive attitude, which in fact could be due to high number of sexually inactive participants, who are expected to be reluctant about the issue. Assessing the knowledge, attitude and usage of EC and the relationship of these factors and some socio-demographic characteristics plays a leading role in public health projects which are aimed to combat maternal mortality through reducing unintended pregnancies. To change attitude towards EC and further increase the level of awareness and usage, collaborated health education and similar studies among health and Media workers are highly recommended. A separate study to assess the level and the type of forced sexual intercourse is also recommended.

## Competing interests

The authors declare that they have no competing interests.

## Authors' contributions

FAA was involved in study conceptualization, data collection, and data analysis, interpretation of results and drafting of the manuscript. KMM participated in designing the study, data analysis, drafting and review of the manuscript. KOP also participated in critical review of the manuscript. BOA helped in revising the manuscript. All authors read and approved the final manuscript.

## Pre-publication history

The pre-publication history for this paper can be accessed here:

http://www.biomedcentral.com/1471-2458/12/110/prepub

## Supplementary Material

Additional file 1 **Questionnaire**. Format to assess the knowledge, attitude and practice towards emergency contraception among Addis Ababa University under graduate female students, Ethiopia. Click here for file
