# Supplementary material for: Assessing knowledge, attitude, and practice of emergency contraception: a cross- sectional study among Ethiopian undergraduate female students
Source: BMC Public Health. 2012 Feb 9;12:110. doi: 10.1186/1471-2458-12-110 (PMC3293041; doi:10.1186/1471-2458-12-110)
Supplement: Additional file 1 — Questionnaire. Format to assess the knowledge, attitude and practice towards emergency contraception among Addis Ababa University under graduate female students, Ethiopia. [file 1471-2458-12-110-S1.DOC]

**Additional file**

**Questionnaire**

**Format to assess the knowledge, attitude and practice towards emergency contraception among Addis Ababa University under graduate female students, Ethiopia**

**Instruction**

1. No need of mentioning once name
2. You are politely requested to give a genuine response
3. Respond by ticking the correct answer
4. Be informed that all of your responses will be kept confidential and you have all the right not to respond if you don’t want to.

**I. Socio-demography**

1. Age ____________

2. In which year of study are you?

1. First year
2. Second year
3. Third year
4. Fourth year
5. Other(specify)

3. Religion

1. Muslim
2. Orthodox Christian
3. Protestant Christian
4. Other (Specify)

4. Ethnicity

1. Amhara
2. Oromo
3. Tigre
4. Other (specify)

5. Which region of Ethiopia have you been living before you joined AAU?

6. What is your department of affiliation?

7. What is your current marital status?

1. Unmarried
2. Married
3. Divorced
4. Widowed

8. How much is the estimated monthly income of your parents?

1. less than 150 birr
2. 150-249 birr
3. 250-499 birr
4. 500-999 birr
5. 1000-1499birr
6. more than 1500birr

9. What is your Mother’s Educational level?

1. Can’t write and read
2. Can write and read
3. Primary school (1-8^th^ grade)
4. Secondary school (9th -12^th^ grade)
5. Higher education

10. What is your Father’s Educational level?

1. Can’t write and read
2. Can write and read
3. Primary school (1-8^th^ grade)
4. Secondary school (9th -12^th^ grade)
5. Higher education

**II. Reproductive history**

1. Are you sexually active (any previous history of sexual intercourse)?

1. Yes

2. No

If the answer for the above question is yes, please answer question number 2 to 4

2. Did you have unprotected sexual intercourse?

1. Yes

2. No

3. How many children do you have?

1. zero
2. one
3. two
4. other(specify)

4. Did you have unintended pregnancies?

1. Yes
2. No

5. If the answer for question number 4 is ‘yes’, how did you become pregnant?

- 1. Contraceptive failure
  2. Forget to take contraceptive
  3. pressure from partner
  4. Forced to have sex
  5. Don’t know about contraceptives
  6. Other( specify)

**III. Knowledge, attitude and practice**

1. Have you ever heard of emergency contraception?

1. Yes

2. No

2. If the answer for the above question is ‘yes’, what was your source of information?

1. Formal education
2. Media
3. Magazines
4. Internet
5. Health facilities
6. Other (specify)

3. When was the first time you heard about emergency contraception?

1. Less than 6 months ago
2. 6-11 months ago
3. 1-5 years ago
4. Before 5 years

4. Do you know where a woman can obtain emergency contraception?

1. Hospital(health center)
2. Social worker( community worker)
3. Private clinic
4. pharmacy
5. supermarket
6. other(specify)
7. It’s impossible to obtain
8. Don’t know

5. Have you heard of any other contraception methods?

1. Yes

2. No

6. If the answer for question number 1 is ‘yes’, what do you think of emergency

contraception?

1. It may cause health problem
2. It may hurt in case it doesn’t work
3. It may result in complications to get pregnant in the future
4. Its use may be illegal
5. It will result in more women suffering from sexually transmitted infections (STI) and even HIV/AIDS.
6. If men know that this method exists then would exert pressure on

Women to use it

1. Some women may use it frequently instead of using regular contraceptive
2. other( specify)
3. I don’t know

7. From what you know about emergency contraception, do you think you would ever use it or recommend it to a friend or relative in case of need?

1. Yes
2. No
3. I am not sure

8. When do you think emergency contraception is important?

- - 1. Post rape
    2. Back up when condom breaks
    3. If oral contraceptive pills(OCP) is forgotten
    4. Other(specify)

9. How long after unprotected sex should emergency contraceptive pill be taken?

1. Immediately after sex
2. within 24 hours
3. within 120 hours /five days
4. within one week
5. at any time before the first day
6. at the next menses
7. Other/specify
8. I don’t know

10. How effective are emergency contraceptive pills in preventing a pregnancy?

1. 99%
2. 75%
3. 50%
4. 40 %
5. Not sure

11. How safe do you think emergency birth control methods are for most women?

1. Very safe
2. Safe
3. Unsafe
4. I don’t know

12. Have you ever used any contraceptive?

1. Yes
2. No

13. Have you ever used emergency contraceptive?

1. Yes
2. No

14. If the answer for question number 13 is ‘yes’, who recommend it for you?

1. friend
2. partner
3. telephone line
4. web page
5. Health professional
6. don’t remember
7. other(specify)

15. If the answer for question number 13 is ‘yes’, what was the reason for using that?

1. No use of other contraception
2. timing miscalculation
3. condom broke or slipped
4. Missed pills
5. you were forced to have sex
6. the withdrawal fail
7. other(specify)
8. I don’t remember
